# Latent Representation-Based Learning Controller for Pneumatic and Hydraulic Dual Actuation of Pressure-Driven Soft Actuators

**DOI:** 10.1089/soro.2022.0224

**Published:** 2024-02-13

**Authors:** Taku Sugiyama, Kyo Kutsuzawa, Dai Owaki, Mitsuhiro Hayashibe

**Affiliations:** Department of Robotics, Graduate School of Engineering, Tohoku University, Sendai, Japan.

**Keywords:** soft robotics, pressure-driven soft actuator, iterative learning control, feedforward neural network, individual deformability compensation, trajectory tracking

## Abstract

The pneumatic and hydraulic dual actuation of pressure-driven soft actuators (PSAs) is promising because of their potential to develop novel practical soft robots and expand the range of soft robot applications. However, the physical characteristics of air and water are largely different, which makes it challenging to quickly adapt to a selected actuation method and achieve method-independent accurate control performance. Herein, we propose a novel LAtent Representation-based Feedforward Neural Network (LAR-FNN) for dual actuation. The LAR-FNN consists of an autoencoder (AE) and a feedforward neural network (FNN). The AE generates a latent representation of a PSA from a 30-s stairstep response. Subsequently, the FNN provides an individual inverse model of the target PSA and calculates feedforward control input by using the latent representation. The experimental results with PSAs demonstrate that the LAR-FNN can meet the requirements of dual actuation control (i.e., accurate control performance regardless of the actuation method with a short adaptation time) with a single neural network. The results suggest that a LAR-FNN can contribute to soft dual-actuation robot development and the field of soft robotics.

## Introduction

Soft actuators have considerable advantages in terms of flexible interactions with their surrounding environments. These features enable soft robots to complete tasks that are difficult for conventional rigid robots,^1,2^ such as safer human–robot interactions, resulting in a wide range of novel applications (e.g., bioinspired soft robots^3^ and soft robotic grippers^4^). A comprehensive review^5^ identified six classes of soft actuators: electrically responsive, magnetically responsive, thermally responsive, photo-responsive, pressure-driven, and explosive-based actuators. Among these types of soft actuators, pressure-driven soft actuators (PSA; Fig. 2) have particularly diverse practical applications (e.g., wearable hand rehabilitation devices^6,7^) owing to their significant advantages in actuation speed, force density, and deformability.^5^

A PSA is driven by various pressurized fluids flowing into the internal chamber. Typical pressurizing fluids include air (pneumatic actuation) and water (hydraulic actuation).^5^ Existing soft robots properly utilize one type of pressurized fluid depending on their applications, for example, hydraulic actuation for soft fish robots^8^ and pneumatic actuation for soft food grippers.^9^ The physical characteristics (e.g., density and viscosity) of air and water are significantly different,^10^ resulting in significant variations in the operating characteristics of pneumatic and hydraulic PSAs. For example, Focchi et al. demonstrated that the stiffness of hydraulic McKibben muscle increases more linearly with increasing internal pressure than that of pneumatic muscle.^11^

In addition, the pneumatic muscle resulted in more accurate dynamic positioning owing to the lower viscosity of air, and the hydraulic muscle was more reactive to load variations, which led to higher robustness against disturbances.^11^ There are also characteristic differences in energy efficiency,^12^ actuation speed, and force generation velocity.^13^ Given these differences, if the pneumatic and hydraulic dual actuation of a PSA is realized, it should expand the range of PSA applications. For example, dual actuation can realize a soft amphibious robot that uses different fluids in the water and ground.

The general characteristics of soft actuators (nonlinearity, hysteresis,^1^ and individual deformability differences^14^) render their control challenging with a simple model-based or model-free controller.^15–18^ Therefore, a learning controller is currently a popular solution for soft actuator control, which has overcome the difficulties in soft actuator control.^19^ A learning controller is also a potential solution for dual actuation control. For practical applications of dual actuation, a controller must realize sufficient control performance to achieve assigned tasks, regardless of the actuation method. The controller also needs to adapt to the diverse surrounding environments of dual-actuation PSAs. A learning controller is expected to automatically learn about and adapt to the significant characteristic differences between pneumatic and hydraulic PSAs and various environments without model derivation and parameter tuning.

Many researchers have developed learning controllers to control soft actuators,^15,19–36^ and achieved precise control performance. The existing learning controllers can be classified into two types depending on the amount of prior model information: a model-based learning controller and a model-free learning controller.^19^ As an example of a model-based learning controller, Tang et al. developed an iterative learning model predictive controller.^31^ This controller gradually improves and fits the approximated initial model to the surrounding environments through an iterative trial-and-error process.

The controller achieved precise trajectory tracking with a root mean square error (RMSE) <0.03 rad. A model-free learning controller does not require prior information. For example, we developed an iterative learning-based feedforward neural network (IL-FNN).^15^ The IL-FNN obtains an inverse model from the control result of an iterative learning controller (ILC). The IL-FNN achieved precise tracking on generalized trajectories with an RMSE of 2.11°. This category also includes a reinforcement learning controller.^36^ The Supplementary Data provides a further literature review.

However, to our best knowledge, no research has addressed a learning controller that can be applied to the dual actuation of PSAs because all the developed learning controllers have to specialize to only one soft actuator. For example, an FNN optimizes its neural network to be equivalent to a control target's model.^15^ The characteristic differences between pneumatic and hydraulic PSAs are too significant to compensate for with one specialized learning controller,^11–13^ despite the generalization ability of the learning controller.

Thus, the existing controller requires re-specialization after every switch of the actuation methods. Training data are essential for learning controllers to re-specialization. Previous studies suggest that the training data recollection will take >3 min^32^ or ∼5 min.^34^ In addition, there is extra time required for retraining. Considering the application of soft robot systems in the real world, interruption of robots' functioning in operation could be critically consequential, rendering a dual-actuation PSA system less practical.

To address the stated issue, a nonspecialized type learning controller will be suitable for dual actuation. For example, suppose a learning controller provides a model of a target PSA based on the PSA's characteristic information source, which can be obtained in a short time (e.g., impulse response). In that case, the controller is expected to quickly adapt to the target PSA (i.e., the actuation method) and the diverse surrounding environment. As a result, the controller will realize precise control performance for both actuation methods, avoiding the function pause.

This study aims to develop a nonspecialized type learning controller for pneumatic and hydraulic dual-actuation PSAs, which has not been addressed in the literature. To this end, we propose a LAtent Representation-based Feedforward Neural Network (LAR-FNN). The LAR-FNN consists of an autoencoder (AE) as a characteristic data extractor and a simple feedforward neural network (FNN) as a feedforward control input generator. The LAR-FNN realizes tracking control for a generalized reference trajectory, receiving a 30-s stairstep response of a PSA. The LAR-FNN was evaluated using an actual PSA.

## Methods

### Architecture of the LAR-FNN

**FIG. 1.**
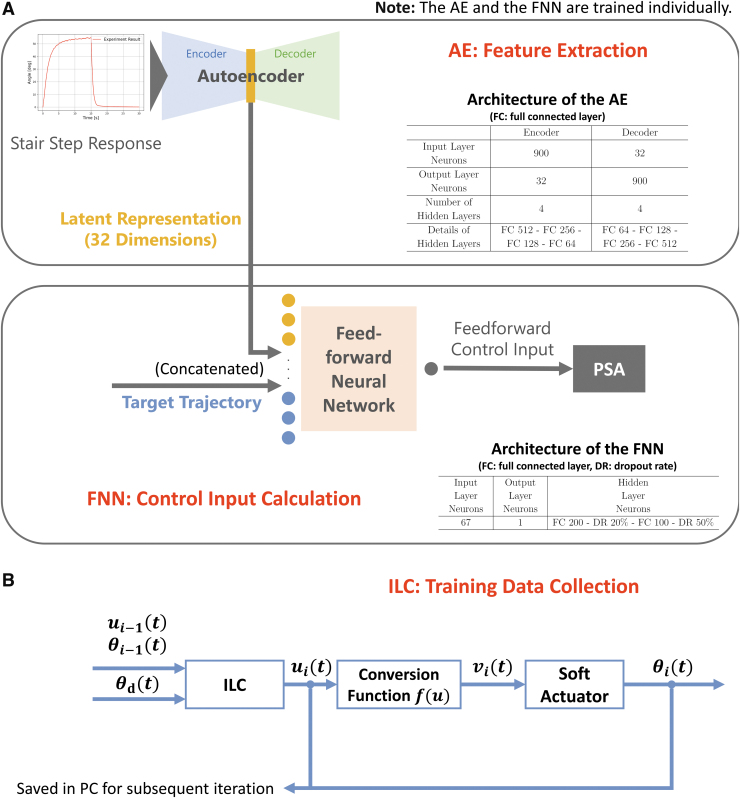
**(A)** Architecture of the proposed LAR-FNN. The LAR-FNN consists of an AE and a FNN. The AE was used to extract features from a step response of a control target. The AE receives a 30-s stairstep response of a PSA and calculates its latent representation. The extracted latent representation represents the parameters of the soft actuator's simplified model (transfer function), which has sufficient dynamic characteristic information. Then, the FNN receives the latent representation and reference trajectory to provide the inverse model for each PSA with different actuation methods. The FNN calculates control input as a feedforward signal. Owing to the extracted latent representations, the FNN can efficiently grasp the control characteristics of the control target to generate an inverse model. **(B)** Process flow of the ILC. The ILC provides a supervised training data set for the FNN. The ILC gradually improves the tracking performance of a control target by repeating the same movement. At *i*th iteration, the ILC first calculates control input $u_{i}\left(t\right)$ from reference trajectory $\theta_{d}\left(t\right)$ and control input and results at the previous iteration ($u_{i-1}\left(t\right),\theta_{i-1}\left(t\right)$). Then, the conversion function converts *u* to *v* to linearize the input–output relation of a soft actuator to stabilize the iterative learning process and improve the control performance. Finally, *v* controls the soft actuator. The Supplementary Data describes the detailed equation and workflow. The control results include information on coping with individual characteristics to realize neat movements. Thus, the FNN can efficiently learn an inverse model of each soft actuator from the training data. AE, autoencoder; FNN, feedforward neural network; ILC, iterative learning controller; LAR-FNN, LAtent Representation-based FNN; PSA, pressure-driven soft actuator.

Figure 1A shows the architecture of the LAR-FNN. First, the AE receives a 30-s stairstep response of a PSA and calculates its latent representation. A latent representation is low-dimensional data extracted from original data, which contains sufficient essential features to reconstruct the original data (e.g., actuation method and individual deformability). Thus, it is expected that the latent representations of the stairstep responses contain the control characteristics of the control target. The FNN receives the latent representation and reference trajectory to provide the inverse model (a model that can generate control input to achieve received control goals) of the PSA and calculate control input as a feedforward signal.

**FIG. 2.**
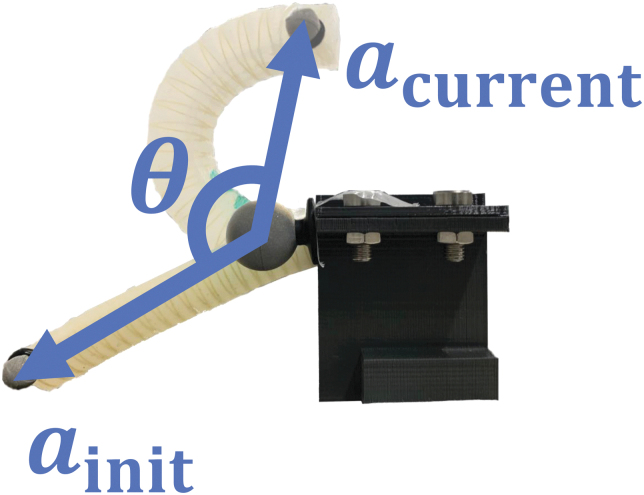
An FRSBA is a type of PSA. The FRSBA was fixed to the *black* fixture on the *right* side. The *gray* spheres on the FRSBA and the fixture are motion capture markers. FRSBA, fiber-reinforced soft bending actuator.

Owing to the extracted latent representations, the FNN can efficiently grasp the control characteristics of the control target to generate an inverse model. By training the FNN to learn inverse models for each latent representation, the FNN calculates the control input for each soft actuator with different actuation methods and characteristics to realize accurate trajectory tracking. Furthermore, an FNN has a generalization ability and can adapt to untrained operating conditions.^34^ Thus, it is expected that the LAR-FNN can calculate the control inputs for soft actuators that are not used for training.

### Architecture of the AE

The AE was used to extract features from a step response of a control target. The AE can perform dimensionality reduction for various types of input data,^37^ including the PSA's nonlinear time-series data with dead time. Note that we did not employ a variational AE because the AE was used for encoding characteristics of the control target, not for a generative task. The input to the AE is a 30-s stairstep response of a control target. The output of the AE is the input stairstep response reconstructed from the latent representation. Two neural networks, an encoder and decoder, comprise the AE. The latent representation is obtained from its bottleneck layer.

The trained AE is expected to extract the factors of differences in step responses (actuation method and individual deformability) into the latent representation. Figure 1A describes the detailed architectures of the encoder and decoder. Each of the encoder's input layer and decoder's output layer had 900 neurons, corresponding to the data length of the 30-s response with a 30-Hz control frequency. The dimensions of the latent representation and the hidden layers of the encoder and decoder were set to 32 through a preliminary experiment described in the Supplementary Data. The mean squared error (MSE) loss function and ADAM optimizer^38^ with a learning rate of 0.001 were used.

**FIG. 3.**
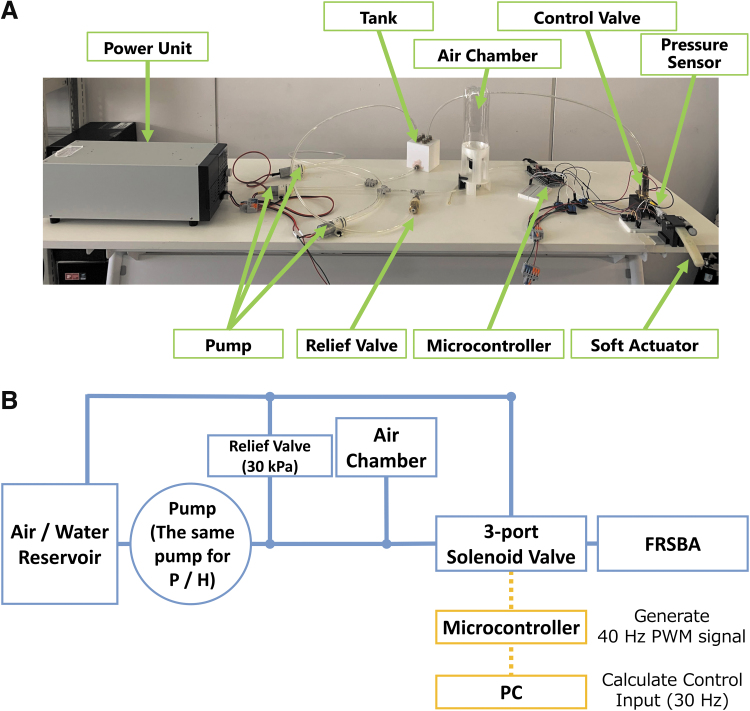
Experimental setup. Three air/water dual-use pump (DSA-2FT-24W; DENSO SANGYO) was parallelly connected to each other and utilized as a pressure source. The three-port solenoid valve (VDW200 series; SMC) regulated the pressure supplied to the FRSBA. The relief valve with a 30-kPa relief pressure regulated the input pressure to the FRSBA (i.e., the pressure upstream of the three-port solenoid valve) at a constant 30 kPa to avoid excessive actuation of the FRSBA. The air chamber reduced the pump's periodic fluid discharge rate variation. The control input was calculated with the PC, and the input was transmitted to the microcontroller (Arduino Mega 2560 R3; Arduino). The microcontroller generated PWM signals, and the PWM signals operated the valve openings to control the FRSBA's internal pressure. A motion capture system (OptiTrack Prime 17W; NaturalPoint) tracked markers on the FRSBA, and the pressure sensor (SSC series; Honeywell) measured its internal pressure. The measured bending angle and the internal pressure were transmitted to the PC. Note that we ensured pneumatic/hydraulic actuation by completely drying the entire system or filling the entire system with water before experiments. **(A)** Photograph of the equipment. **(B)** Diagram of the equipment. PWM, pulse-width modulation.

The stairstep response was used to extract the latent representation because it was suitable for controlling the PSA with the experimental setup described in Figure 3. As shown in Supplementary Figure S1, the stairstep response primarily contained information on the low-frequency domain. Pneumatic PSAs have been actuated rapidly with appropriate pneumatic components.^17,39–42^ However, in this study, the pump employed for the experiments actuated the pneumatic PSAs at a low frequency of <2 Hz. Consequently, high-frequency domain data were unnecessary for PSA control in the experiments, as high-frequency noise can negatively affect the AE training. Also, the stairstep response contains characteristic information on both the increasing and decreasing responses. Furthermore, a quick adaptation to the actuation method can be realized by terminating the measurement in 30 s, which provides plenty of time for the response to be in a steady state.

### Architecture of the FNN

The FNN provides an inverse model and calculates the control input for each soft actuator according to the latent representation received. The FNN receives concatenated data of the latent representation and control target values. For the control input calculation, the control target value input is time-series data around target time *t*, which considers a target PSA's slow response to internal pressure change.^1^ The FNN then produces a control input at the target time *t*. The control target value at t<0 and $t_{end}<t$ was zero, where $t_{end}$ indicates the control termination time.

Figure 2A presents the detailed architecture of the FNN. The input layer neurons were set to 67 with 17 empirically determined samples around the target time *t*. Note that 67 is the sum of the latent representation dimension (32), a sample at t(1), and 17 samples before and after t(34). The output layer had one neuron, and the number of hidden layers was two, with dropout rates of 20% and 50%. The MSE loss function and ADAM optimizer with a learning rate of 0.001 were utilized.

The FNN is trained using supervised learning. Similar to the IL-FNN, the simple trajectory tracking results of an actual soft actuator obtained using an ILC are utilized as a training data set. The ILC gradually improves the tracking performance of a control target by repeating the same movement.^43^ Through this iterative process carried out for each soft actuator, the ILC can automatically generate a suitable control input for each of them without laborious parameter tuning. The control results include information on coping with individual characteristics to realize neat movements, and the FNN can efficiently learn each inverse model from the training data.^15^ Figure 1B shows the process flow of the ILC.

## Experimental Setup

### Pressure-driven soft actuator

In this study, a fiber-reinforced soft bending actuator (FRSBA) that bends upon pressurization was utilized as a control target (Fig. 2). FRSBAs have been used in various applications, such as soft rehabilitation devices.^6^ The supplementary document describes the operating principle and fabrication materials of FRSBAs.

The control input for the FRSBA was the pulse-width modulation duty cycle (PWM D.C.) of a valve that was connected to it. The PWM D.C. controlled a ratio of fluids that flowed in to/out from an FRSBA and actuated the FRSBA. An FRSBA was fixed to ensure uniform experimental conditions. The angle θ (Fig. 2) between the initial and current fixing-to-tip vectors ($a_{init}$ and $a_{current}$) was controlled; it was measured using Equation (1).

(1) $$\theta =\arccos\left(\frac{a_{init}\cdot a_{current}}{\left|a_{init}\right|\left|a_{current}\right|}\right).$$

### Experimental equipment

Figure 3 shows the experimental setup. The same experimental setup was used for both pneumatically and hydraulically actuated FRSBAs. The pulse-width modulation (PWM) frequency was empirically set to 40 Hz. The control frequency was 30 Hz, which was higher than the FRSBA actuation speed (1–2 Hz at maximum^6^) and lower than the PWM frequency.

### Training data collection for the LAR-FNN

**FIG. 4.**
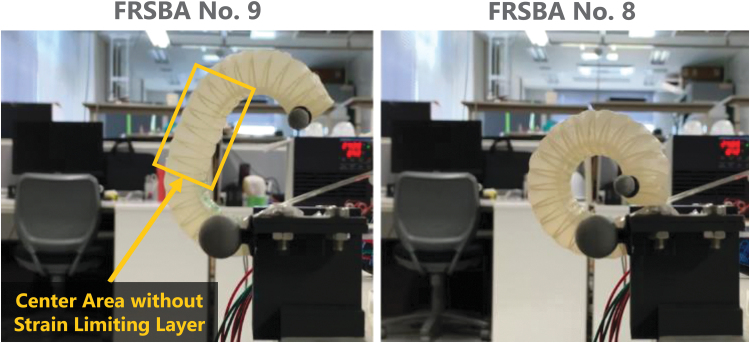
Photographs of FRSBA No. 8 and No. 9 at each maximum bending angle. Supplementary Video S2 provides step responses of FRSBA No. 8 and No. 9. As described in this figure, the center area of FRSBA No. 9 extends upon pressurization. FRSBA No. 9 has no strain limiting layer in its longitudinal center. This structure allows its central part to extend during pressurization because both flat and curved surfaces extend to the same length (see also the Supplementary Data). In this way, the movements of FRSBA No. 9 are significantly different from those of the other FRSBAs.

A data augmentation technique was employed to collect the training data for the AE because it was unrealistic to fabricate many FRSBAs for a large amount of data. First, 6-, 10-, 15-, 30-, 60-, 90-, and 150-s stairstep responses were obtained from one pneumatic or hydraulic FRSBA. Note that the pump and the relief valve with a 30-kPa relief pressure (Fig. 4) maintained input pressure to the FRSBAs at a constant 30 kPa. Next, upsampling or downsampling was performed so that all the collected step responses became 30 s. For upsampling, two sampling points were linearly interpolated with three (6 s), two (10 s), or one (15 s) additional sampling points.

For the downsampling process, the first sampling data were extracted from a set of two (60 s), three (90 s), or five (150 s) sampling data points from the beginning of the step responses. After performing the up- or downsampling, the processed data were augmented by multiplying the magnitude (×0.5, ×0.6, …, ×1.0, ×1.2, …, ×2.0) and dead time addition (+0.0 s, +0.1 s, … +0.9 s). These data augmentation processes supplied 770 samples of different stairstep responses from a single pneumatic or hydraulic FRSBA.

The FRSBA was controlled to track single-period sine trajectories of various amplitudes and frequencies (Supplementary Fig. S2) to collect adequate data for the FNN. The results of all 15 iterations were used to diversify the training data sets. The learning gains (see Equation (S1) in the Supplementary Data) were set through a trial-and-error process as $\Gamma_{1}=9.6,\Gamma_{2}=0.03$ for pneumatic actuation and $\Gamma_{1}=16.0,\Gamma_{2}=0.05$ for hydraulic actuation, not to cause huge FRSBA oscillations and to converge the iterative learning process as quickly as possible. After finishing all 15 iterations, the control results were processed using a low-pass filter with a cutoff frequency of 10 Hz to reduce noise.

## Experimental Results

**FIG. 5.**
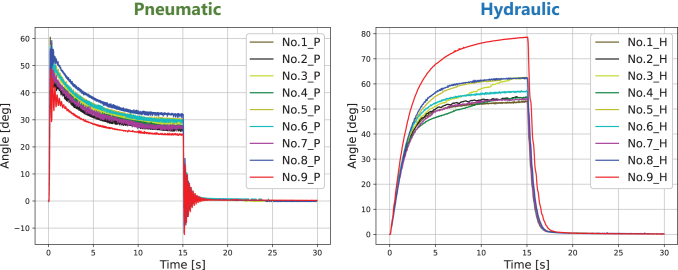
Stairstep responses of all FRSBAs utilized in this study. P/H in the legend indicates that the corresponding FRSBA is pneumatically or hydraulically actuated.

A single LAR-FNN was trained with one data set that included data from five FRSBAs actuated both pneumatically and hydraulically. The data set included validation data from two FRSBAs actuated pneumatically and hydraulically. The trained LAR-FNN was then evaluated with two pneumatically and hydraulically actuated FRSBAs, which were not included in the data set. The movements of one of the FRSBAs for the evaluation (No. 9) significantly differed from those of the other FRSBAs (Fig. 4), owing to the internal structural differences. Thus, FRSBA No. 9 was used for the evaluation to investigate the versatility limitation of the LAR-FNN. Table 1 lists the FRSBA parameters. Figure 5 shows the stairstep responses to the same 50% PWM D.C. for each FRSBA. We observed significant differences in the transient and steady responses between the two actuation methods and between individual FRSBAs with the same actuation method.

**Table 1.** Parameters of the Fiber-Reinforced Soft Bending Actuators

No.	Used for	Length, mm	The radius of the semicircle, mm
1	Training	88	19
2	Training	87	19
3	Training	87	19
4	Training	87	19
5	Training	88	19
6	Training (validation)	87	19
7	Training (validation)	88	19
8	Evaluation	87	19
9	Evaluation (different structure)	92	19

### Evaluation of the AE

**FIG. 6.**
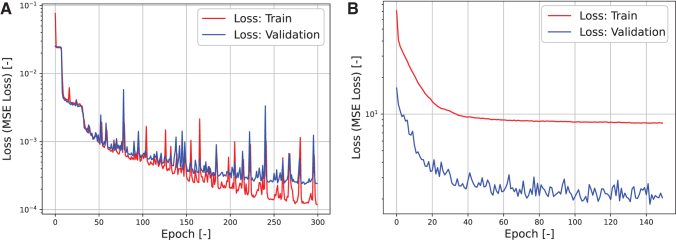
Training curves of the **(A)** AE, **(B)** FNN. These training curves indicate that no overfitting occurred during the AE and FNN training.

**FIG. 7.**
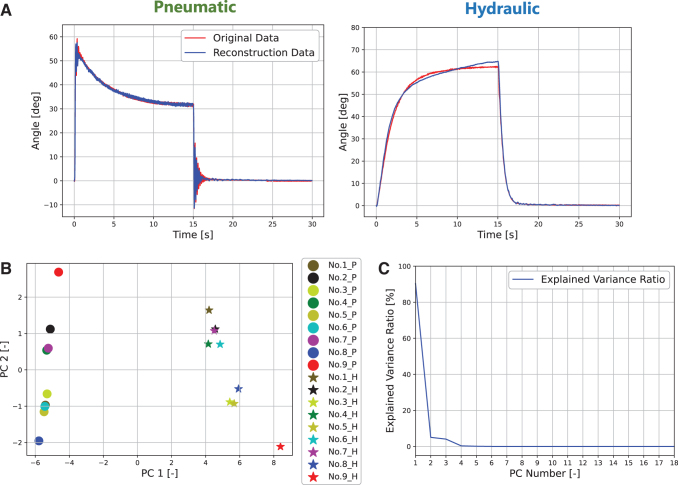
**(A)** Original stairstep responses and AE-reconstructed responses (pneumatic and hydraulic FRSBA No. 8.). **(B)** Principal component analysis results for the extracted latent representations. Only principal components PC1 and PC2 are plotted as the explained variance ratio was >95% with these two. The *circle* markers indicate pneumatic actuation, and the *star* markers indicate hydraulic actuation. The marker colors of the FRSBA correspond to the colors in Figure 5. P/H in the legend indicates that the corresponding FRSBA is pneumatically or hydraulically actuated. **(C)** Explained variance ratio.

The AE training was repeated for 300 epochs. Figure 6A shows the training curve of the AE, which indicates that no overfitting occurred. Figure 7A compares the original stairstep response of FRSBA No. 8 with the response reconstructed by the decoder from the latent representation. See Supplementary Figure S3 for the results of the other FRSBAs. The AE correctly reconstructed the received inputs, which means that the latent representation contained vital core data on the FRSBA's characteristics, which allowed the original response to be reconstructed.

**FIG. 8.**
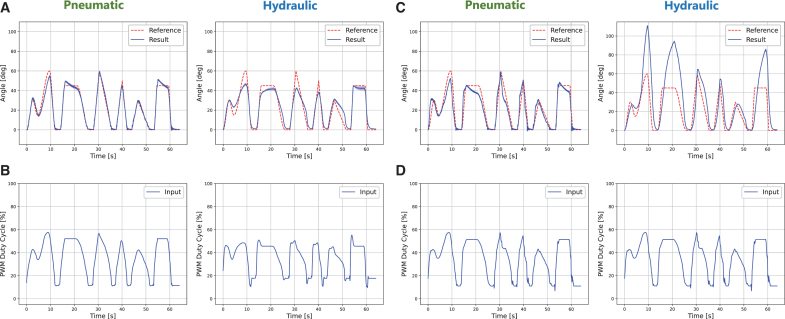
Results of generalized trajectory tracking with FRSBA No. 8. The experiments were repeated five times with the same calculated control input. The *solid line* indicates the average value of five trials, and the light-colored band indicates the standard deviation. **(A)** Results with the LAR-FNN. Supplementary Video S1 provides the movie of the control. Slight oscillation was observed with pneumatic actuation because the three-port solenoid valve and the FRSBA were too close for air with lower viscosity. Note that the vibration was not because of the LAR-FNN, and the corresponding control inputs were always stable. **(B)** Control inputs corresponding to the LAR-FNN control. **(C)** Results with the IL-FNN. **(D)** Control inputs corresponding to the IL-FNN control. Note that the same control input was used to control both pneumatic and hydraulic FRSBAs because the IL-FNN cannot change control inputs based on a latent representation such as the LAR-FNN. IL-FNN, iterative learning-based feedforward neural network.

Figure 8B and C show the results and the explained variance ratio of a principal component analysis (PCA) for the latent representations. As shown in Figure 7B, the latent representation accurately captured the characteristic information of the pneumatic and hydraulic FRSBAs. In addition, the positions of the marks varied even within the same actuation method group, indicating that the individual characteristic differences of the FRSBAs are included in the latent representations.

### Evaluation of the performance of the LAR-FNN

The FNN training was repeated for 150 epochs. Figure 6B shows the FNN training curve, which indicates that no overfitting occurred. For the evaluation, the same LAR-FNN was used to control both the pneumatic and hydraulic FRSBAs using different stairstep response inputs to the AE. A LAR-FNN without the AE component, which had the same architecture as the IL-FNN, was also trained. The training was conducted in the same manner as that of the LAR-FNN to evaluate the latent representation contributions to the LAR-FNN's capability. Supplementary Figure S4 shows the architecture of the IL-FNN.

Figure 8 and Table 2 show the complex trajectory tracking results of the LAR-FNN and IL-FNN with FRSBA No. 8. Both FNN evaluations used the same reference trajectory. The control results with different reference trajectories are shown in the Supplementary Data (Supplementary Figs. S5 and S7 and Supplementary Tables S1 and S3). As shown in Figure 8C and Table 2, the control performance of the IL-FNN was not accurate for both actuation methods due to the identical actuation method-independent control input (Fig. 8D).

**Table 2.** Average Root Mean Square Error of the Generalized Trajectory Tracking Task with Fiber-Reinforced Soft Bending Actuator No. 8

	RMSE, °	
	Pneumatic	Hydraulic
LAR-FNN	2.84 ± 0.09	5.52 ± 0.21
IL-FNN	6.46 ± 0.05	20.88 ± 0.25

IL-FNN, iterative learning-based feedforward neural network; LAR-FNN, LAtent Representation-based Feedforward Neural Network; RMSE, root mean square error.

However, as shown in Figure 8A and Table 2, both pneumatic and hydraulic actuation achieved accurate control with the LAR-FNN. Figure 8B shows that the LAR-FNN generated different control inputs for each actuation method to track the same trajectory. Owing to these latent representation-based control inputs, the FRSBA that was not used for training followed the reference trajectory, which had a significantly different shape from the training data set. Notably, triangular and square trajectories were realized even though the training data only included single-period sine trajectory tracking results.

### LAR-FNN versatility in an ultimate scenario

**FIG. 9.**
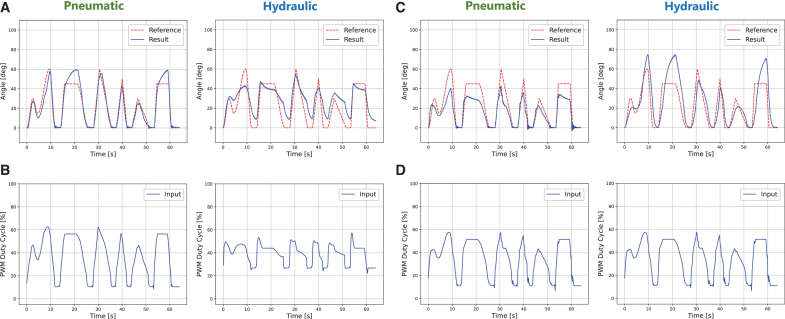
Results of generalized trajectory tracking with FRSBA No. 9. Note that FRSBA No. 9 had a considerably different internal structure and movements from those of the other FRSBAs (see Fig. 4). The experiments were repeated five times with the same calculated control input. The *solid line* indicates the average value of five trials, and the light-colored band indicates the standard deviation. **(A)** Results with the LAR-FNN. **(B)** Control inputs corresponding to the LAR-FNN control. **(C)** Results with the IL-FNN. **(D)** Control inputs corresponding to the IL-FNN control. The same control input as that shown in Figure 8D was used for both actuation methods.

The LAR-FNN and the IL-FNN, which were described in the previous section, controlled FRSBA No. 9, an almost different soft actuator from that used during training. The reference trajectory was the same as that shown in Figure 8. Figure 9 and Table 3 describe the complex trajectory tracking results with FRSBA No. 9 of the LAR-FNN and IL-FNN, respectively. Note that the control results with different reference trajectories are shown in the Supplementary Data (Supplementary Figs. S6 and S8 and Supplementary Tables S2 and S4). Figure 9A shows that the tracking accuracy with the LAR-FNN was less accurate than that with FRSBA No. 8 for both actuation methods. In contrast, as demonstrated in Figure 9C, the control performance with the IL-FNN was worse than that with the LAR-FNN for both actuation methods.

**Table 3.** Average Root Mean Square Error of the Generalized Trajectory Tracking Task with Fiber-Reinforced Soft Bending Actuator No. 9

	RMSE, °	
	Pneumatic	Hydraulic
LAR-FNN	5.73 ± 0.13	10.88 ± 0.56
IL-FNN	10.29 ± 0.12	13.39 ± 0.32

## Discussion

Figure 5 demonstrates that the same step input induces considerably different step responses between pneumatic and hydraulic FRSBAs. Nevertheless, as shown in Figure 9A and B and Table 2, the LAR-FNN successfully compensated for the sizeable characteristic differences. The LAR-FNN achieved precise generalized trajectory tracking for both actuation methods, only receiving the 30-s stairstep response acquired in a short time. In addition, Figure 9C and D demonstrate the effectiveness of the latent representation in quickly adapting to the significant characteristic differences and achieving precise control performance regardless of the actuation method. Owing to the use of latent representations, the LAR-FNN quickly adapts to a control target's characteristics, generating different control inputs for each actuation method, unlike the IL-FNN (Fig. 9B, D). These results demonstrate the capability and generalization ability of the LAR-FNN to satisfy the requirements for the control of dual-actuation PSAs.

**FIG. 10.**
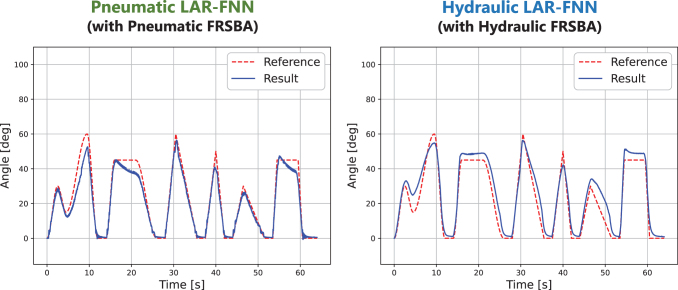
Results of generalized trajectory tracking with the Pneumatic LAR-FNN and the Hydraulic LAR-FNN. The Pneumatic LAR-FNN and the Hydraulic LAR-FNN were trained with a data set that only included data from pneumatically and hydraulically actuated FRSBAs, respectively. We controlled pneumatically actuated FRSBA No. 8 with the Pneumatic LAR-FNN and hydraulically actuated FRSBA No. 8 with the Hydraulic LAR-FNN. The reference trajectory was the same as that shown in Figs. 8 and 9. The control results with a single period sine trajectory are shown in the Supplementary Data (Supplementary Fig. S11). The experiment was conducted once. The other experimental setups (e.g., the experimental equipment, training, and data collected for the data sets) were the same as those with the LAR-FNN.

Table 2 shows that pneumatic actuation resulted in more accurate control with a lower RMSE, although satisfactory control accuracy was also achieved with hydraulic actuation. The limitation of the experiment setup and the physical characteristics of air and water caused this result. Figure 10 and Supplementary Figure S11 and Table 4 show the control results with another LAR-FNN, a Pneumatic LAR-FNN and a Hydraulic LAR-FNN, which was intentionally specialized to pneumatic FRSBAs and hydraulic FRSBAs.

**Table 4.** RMSE of the Trajectory Tracking Tasks with the LAR-FNN, the Pneumatic LAR-FNN, and the Hydraulic LAR-FNN

	RMSE, °			
	Complex trajectory		Sine trajectory	
	Pneumatic	Hydraulic	Pneumatic	Hydraulic
LAR-FNN	2.84 ± 0.09	5.52 ± 0.21	1.94 ± 0.11	5.30 ± 0.43
Pneumatic LAR-FNN	4.23	—	3.30	—
HydraulicLAR-FNN	—	5.44	—	6.75

As demonstrated in Table 4, there was no accuracy improvement even if the LAR-FNN specialized to each actuation method. This result suggests that the decreased control accuracy with the hydraulic actuation is not due to the limitation of the LAR-FNN but the physical characteristics of air and water and the experimental setup. The literature has pointed out that pneumatic actuation results in more accurate dynamic positioning owing to the lower viscosity of air.^11^ Also, tubes with a wider diameter would benefit from feeding water more smoothly and actuating hydraulic FRSBA more rapidly, resulting in more accurate control of hydraulic FRSBAs.

The control performance of the LAR-FNN with FRSBA No. 9 was not as accurate as that with FRSBA No. 8. As shown in Figure 7B, PC1 and PC2 of FRSBA No. 9 (red markers) were far away from the areas covered with the FRSBAs used in training. By contrast, PC1 and PC2 of FRSBA No. 8 (blue markers), which the LAR-FNN precisely controlled, were inside or close to the areas. In this way, Figure 7B shows that the characteristics of FRSBA No. 9 were considerably different from those that the LAR-FNN had learned. FRSBA No. 9, which had an unlearned structure, was outside the areas where LAR-FNN could achieve characteristic difference compensation and accurate control.

This result suggests the versatility limitation of the LAR-FNN under the ultimate scenario (i.e., control of a soft actuator with an unlearned structure). However, considering that the control input consisted of open-loop signals, the LAR-FNN achieved satisfactory accuracy even in the ultimate scenario. In addition, as shown in Figure 9A and C, latent representations are still effective for characteristic difference compensation. Thus, more diverse training data can enlarge the area and significantly improve the capability and versatility of the LAR-FNN.

The proposed feedforward LAR-FNN achieved precise control performance for both pneumatic and hydraulic actuation, quickly adapting to the selected actuation method while only receiving 30-s stairstep responses. Furthermore, considering the experimental results, quick adaptation to various surrounding environments will be possible with the LAR-FNN. Unlike specialized-type learning controllers, the LAR-FNN can quickly adapt to each actuation method after switching, thereby satisfying the requirements for dual actuation control.

In addition, the capability of the LAR-FNN to control both pneumatic and hydraulic PSAs can be applied to PSAs that are not actuated with a fully pneumatic or hydraulic system (i.e., actuation with a mixture of air and water). Previous studies have indicated that a partially pneumatic or hydraulic system leads to different actuation speeds and compliance characteristics.^13,44^ The application of the LAR-FNN can contribute to the development of practical soft robots with dual actuation mechanisms by enabling accurate control with a short adaptation time, regardless of the actuation method. Thus, the proposed LAR-FNN should realize novel dual-actuation soft robots, thereby expanding the range of PSA applications. In addition, the LAR-FNN should contribute to the field of soft robotics because it can compensate for individual deformability differences and secular changes in PSAs and different types of soft actuators.

As a future direction, feedback term augmentation to the LAR-FNN will be an important issue to be discussed. The LAR-FNN is an entirely open-loop controller. Thus, practical applications of the LAR-FNN will require disturbance-reduction techniques. Employing a long short-term memory instead of the FNN will also be beneficial for the length variation of an input time sequence. For future applications, a quantitative evaluation of the performance of the LAR-FNN in adapting to various surrounding environments and the evaluation with an actual dual actuation soft robot will be helpful. Also, the evaluation of the LAR-FNN for a high-frequency domain^42^ should be investigated.

## Conclusion

This study proposes a LAR-FNN, which consists of an AE and an FNN. The LAR-FNN provides the individual inverse model and control input for each PSA only from the PSA's 30-s stairstep response. Then, the FNN provides the individual inverse model and control input to the target PSA using the latent representation. The evaluation with FRSBAs demonstrated the capability and versatility of the LAR-FNN. The single LAR-FNN achieved precise generalized trajectory tracking for both pneumatic and hydraulic FRSBAs that were not used for the LAR-FNN training.

The proposed nonspecialized type learning controller can satisfy the requirements of dual actuation control, realizing accurate control performance regardless of the actuation method, with a short adaptation time. Thus, the LAR-FNN can solve the dual-actuation problem by achieving maximum control performance for each actuation method with a single neural network. The LAR-FNN can contribute to soft dual-actuation robot development and the field of soft robotics, compensating for the sizeable characteristic differences of soft actuators from the characteristic information sources acquired in a short time.

## Supplementary Material

Supplemental data

Supplemental data

Supplemental data
