# Supplementary material for: Latent Representation-Based Learning Controller for Pneumatic and Hydraulic Dual Actuation of Pressure-Driven Soft Actuators
Source: Soft Robot. 2024 Feb 13;11(1):105–17. doi: 10.1089/soro.2022.0224 (PMC10880272; doi:10.1089/soro.2022.0224)
Supplement: Supplemental data [file Supp_DataS1.pdf]

Latent Representation-based Learning Controller for Pneumatic and Hydraulic Dual Actuation of Pressure-driven Soft Actuators – Supplementary Material

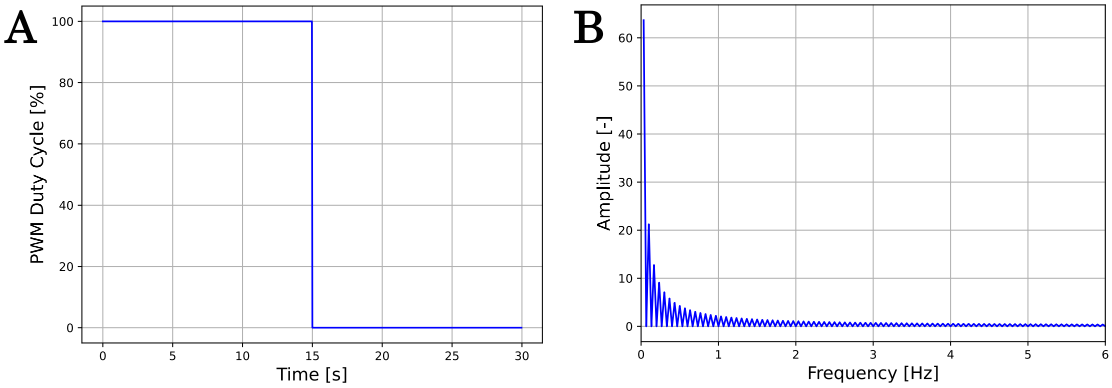

Figure S1. Fourier transform of the 30-seconds stairstep input. (A) Original 30-second stairstep input. (B) Fourier transform result. The stairstep response does not have information on the high-frequency domain of more than 2 Hz.

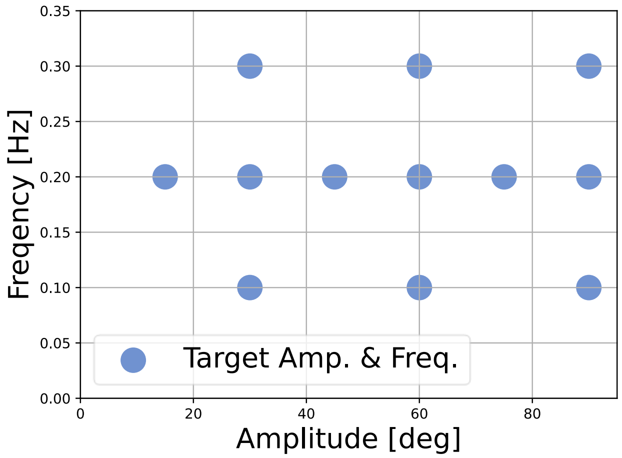

Figure S2. Set of amplitudes and frequencies of the ILC target trajectories for the FNN training data collection.

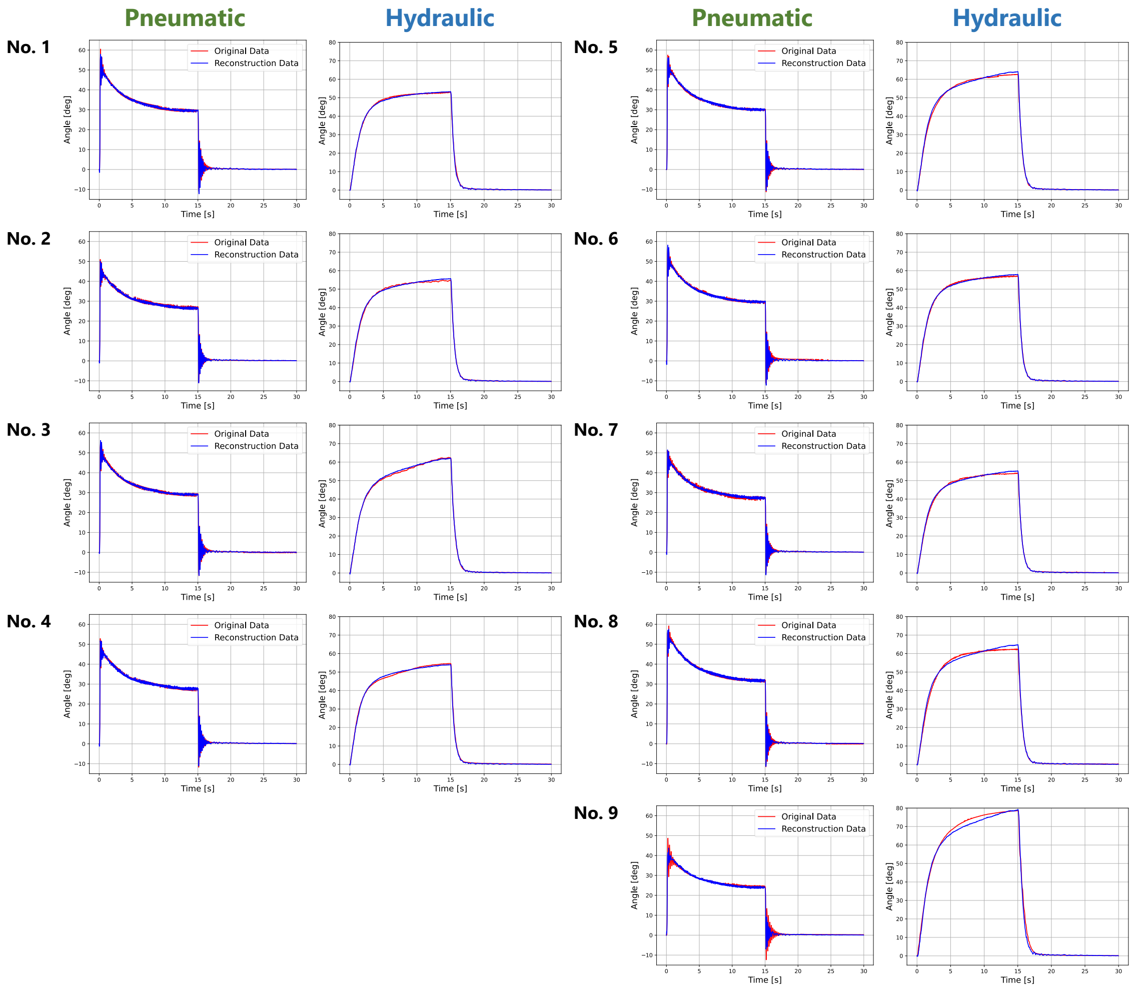

Figure S3. FRSBAs' original stairstep responses and responses generated by the AE. The AE correctly reconstructed received responses for all utilized FRSBAs, which means latent representations contained important and core data on FRSBA's characteristics to decode the original response.

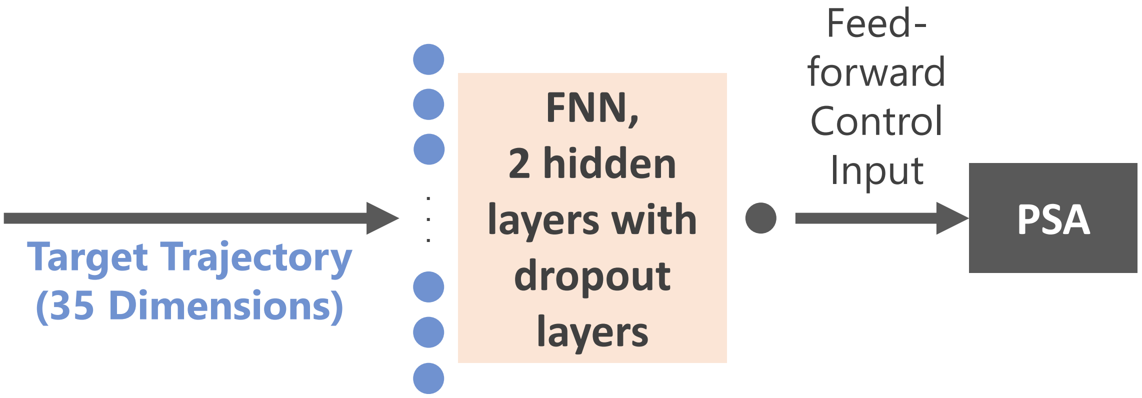

Figure S4. Architecture of the IL-FNN utilized in the experiment. The number of the input layer neurons was 35, and the dataset did not include latent representation data. The other architecture (e.g., the control target, the training data collection with the ILC) and experimental setup were the same as the LAR-FNN.

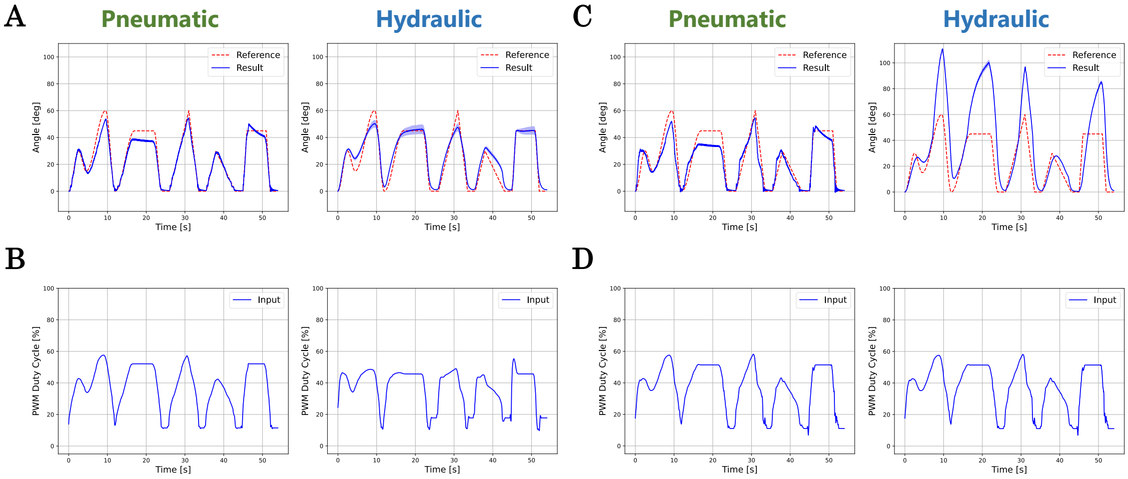

Figure S5. Evaluation of the LAR-FNN with FRSBA No.8 and a different reference trajectory. Table S1 lists corresponding RMSEs. The experiments were repeated five times with the same calculated control input. The solid line indicates the average value of five trials, and the light-colored band indicates the standard deviation. (A) Results with the LAR-FNN. (B) Control inputs corresponding to the LAR-FNN control. (C) Results with the IL-FNN. (D) Control inputs corresponding to the IL-FNN control. Note that the same control input was utilized to control both pneumatic and hydraulic FRSBAs because the IL-FNN can not change control inputs according to a latent representation like the LAR-FNN.

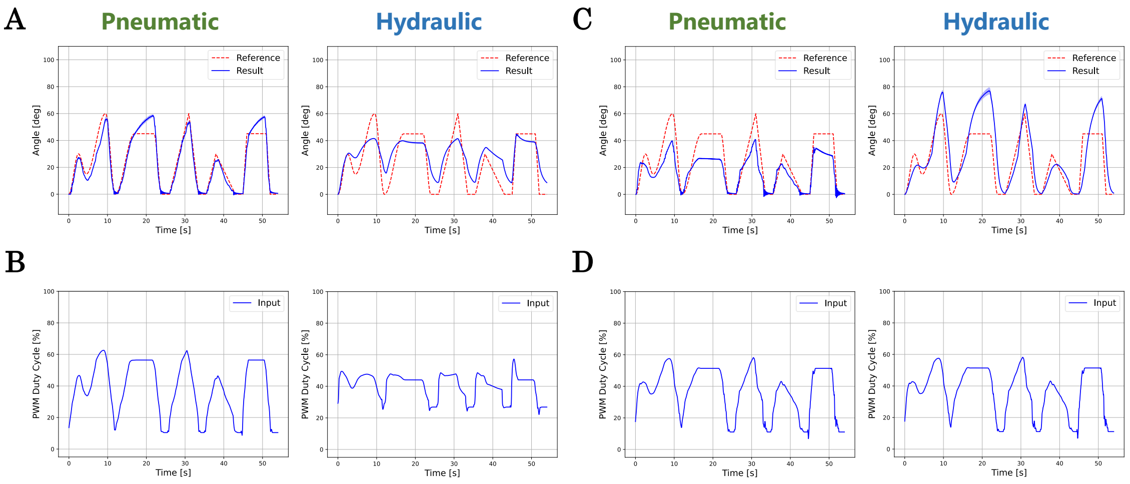

Figure S6. Evaluation of the LAR-FNN with FRSBA No.9 and a different reference trajectory. Table S2 lists corresponding RMSEs. The experiments were repeated five times with the same calculated control input. The solid line indicates the average value of five trials, and the light-colored band indicates the standard deviation. (A) Results with the LAR-FNN. (B) Control inputs corresponding to the LAR-FNN control. (C) Results with the IL-FNN. (D) Control inputs corresponding to the IL-FNN control. The same control input as that shown in Fig. S5(D) was used for both actuation methods.

Table S1. Average RMSE of the trajectory tracking task with FRSBA No. 8, described in Fig. S5

	RMSE [°]	
	Pneumatic	Hydraulic
LAR-FNN	4.17 ± 0.37	5.26 ± 0.32
IL-FNN	7.17 ± 0.23	24.59 ± 0.80

Table S2. Average RMSE of the trajectory tracking task with FRSBA No. 9, described in Fig. S6

	RMSE [°]	
	Pneumatic	Hydraulic
LAR-FNN	5.40 ± 0.17	10.82 ± 0.07
IL-FNN	11.47 ± 0.19	14.84 ± 0.73

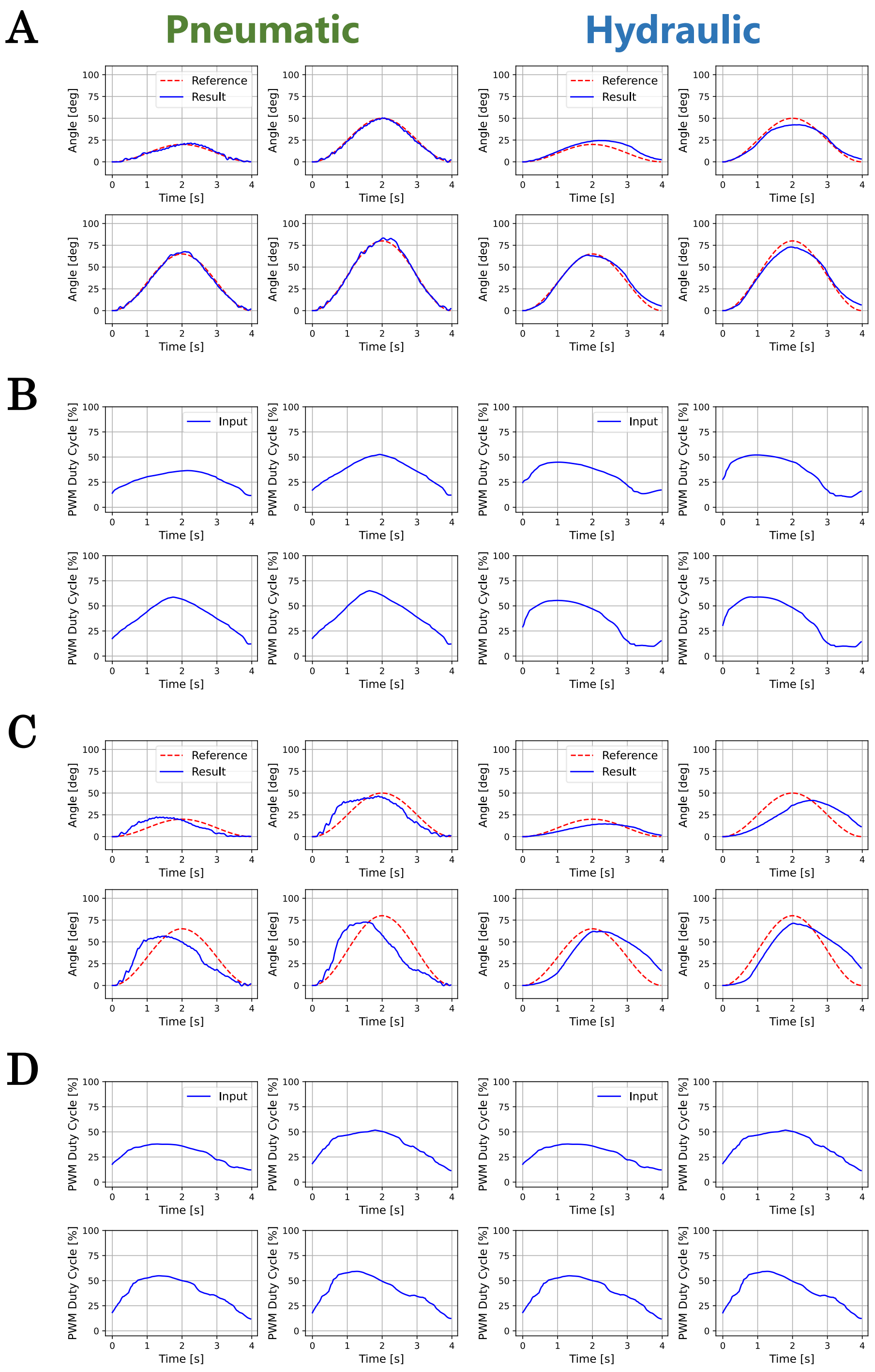

Figure S7. Evaluation of the LAR-FNN with FRSBA No.8 and sine reference trajectories. Note that the amplitudes and frequencies of the sine trajectories differed from those utilized in training data collection (Fig. S2). Table S3 lists corresponding RMSEs. The experiments were repeated three times with the same control input. The solid line indicates the average value of five trials, and the light-colored band indicates the standard deviation. (A) Results with the LAR-FNN. (B) Control inputs corresponding to the LAR-FNN control. (C) Results with the IL-FNN. (D) Control inputs corresponding to the IL-FNN control. Note that the same control input was utilized to control both pneumatic and hydraulic FRSBAs because the IL-FNN can not change control inputs according to a latent representation like the LAR-FNN.

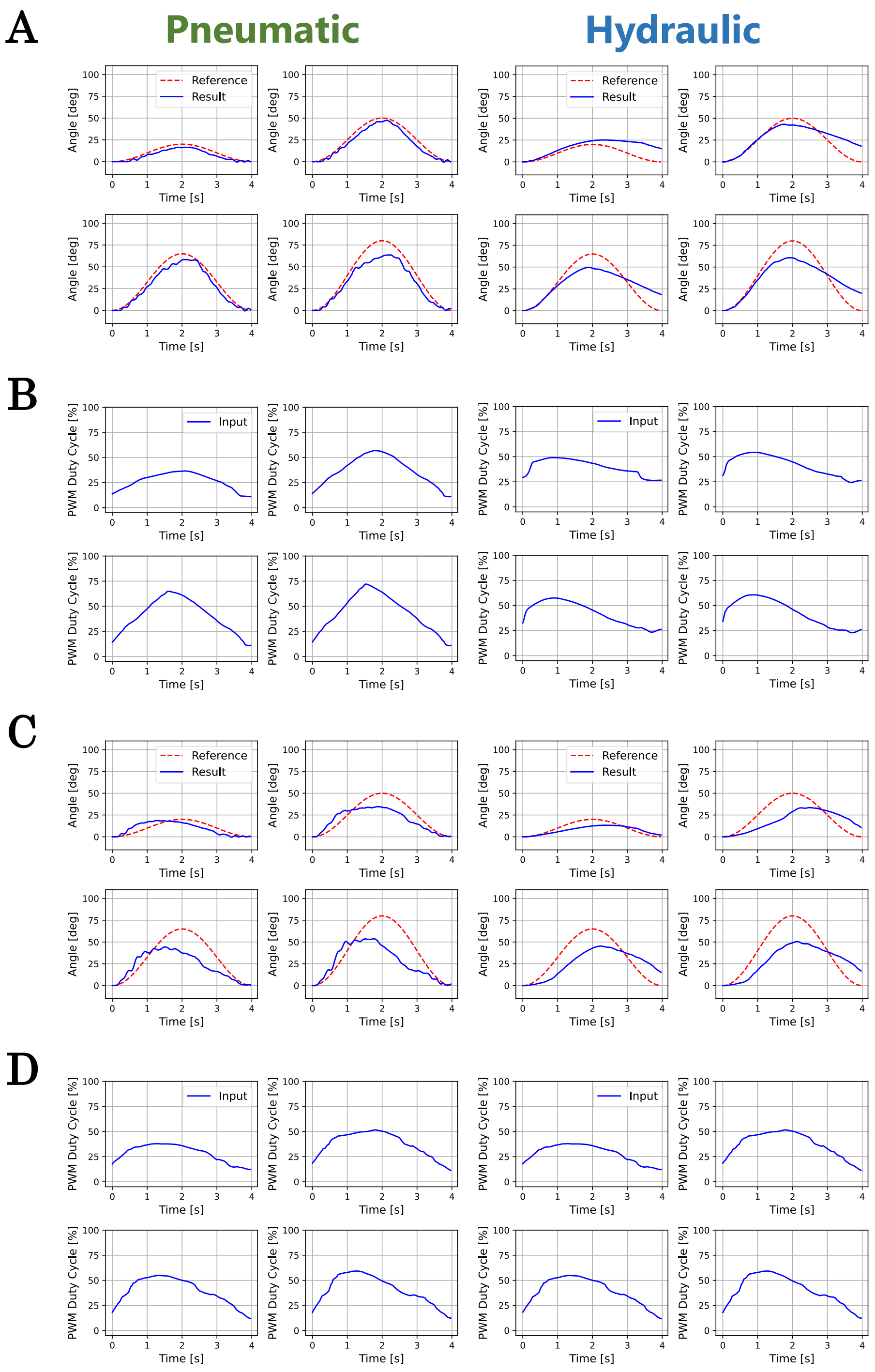

Figure S8. Evaluation of the LAR-FNN with FRSBA No.9 and sine reference trajectories. Note that the amplitudes and frequencies of the sine trajectories differed from those utilized in training data collection (Fig. S2). Table S4 lists corresponding RMSEs. The experiments were repeated three times with the same control input. The solid line indicates the average value of five trials, and the light-colored band indicates the standard deviation. (A) Results with the LAR-FNN. (B) Control inputs corresponding to the LAR-FNN control. (C) Results with the IL-FNN. (D) Control inputs corresponding to the IL-FNN control. The same control input as that shown in Fig. S7(D) was used for both actuation methods.

Table S3. Average RMSE of the sine trajectory tracking tasks with FRSBA No. 8, described in Fig. S7

	Pneumatic				Hydraulic			
Amplitude [°]	20	50	65	80	20	50	65	80
RMSE [°] (LAR-FNN)	1.43 ± 0.02	1.53 ± 0.16	1.71 ± 0.15	2.13 ± 0.16	4.69 ± 0.12	3.86 ± 0.08	3.11 ± 0.11	4.74 ± 0.08
RMSE [°] (IL-FNN)	4.06 ± 0.04	13.22 ± 0.03	14.75 ± 0.08	15.52 ± 0.005	5.32 ± 0.09	8.84 ± 0.17	13.94 ± 0.13	19.77 ± 0.20

Table S4. Average RMSE of the sine trajectory tracking tasks with FRSBA No. 9, described in Fig. S8

	Pneumatic				Hydraulic			
Amplitude [°]	20	50	65	80	20	50	65	80
RMSE [°] (LAR-FNN)	3.07 ± 0.15	4.59 ± 0.19	5.68 ± 0.12	10.23 ± 0.07	9.44 ± 0.17	8.92 ± 0.09	11.34 ± 0.07	13.02 ± 0.12
RMSE [°] (IL-FNN)	4.71 ± 0.01	10.33 ± 0.04	16.11 ± 0.07	21.35 ± 0.06	4.55 ± 0.06	14.94 ± 0.11	16.49 ± 0.15	20.82 ± 0.32

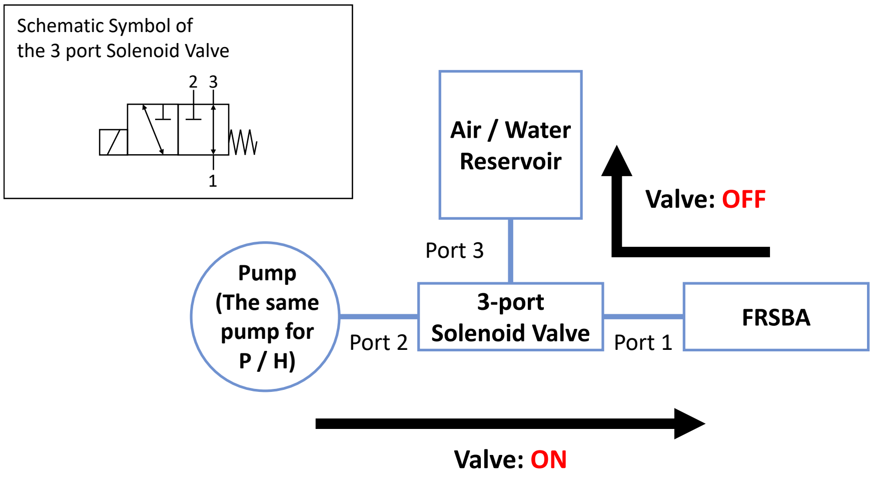

Figure S9. The operation of the 3-port solenoid valve and its schematic symbol. The black arrows indicate fluid flow. The valve connects port 1 (the FRSBA) and port 2 (the pumps) when it is on and connects port 1 (the FRSBA) to port 3 (the air/water reservoir) when it is off.

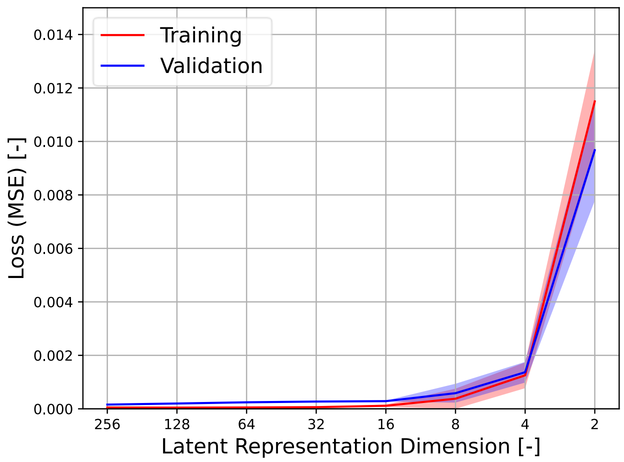

Figure S10. Training loss and validation loss of the LAR-FNN with various latent representation dimensions. The LAR-FNN training was repeated three times with different LAR-FNN instances. The solid line indicates the average value of three trials, and the light-colored band indicates the standard deviation.

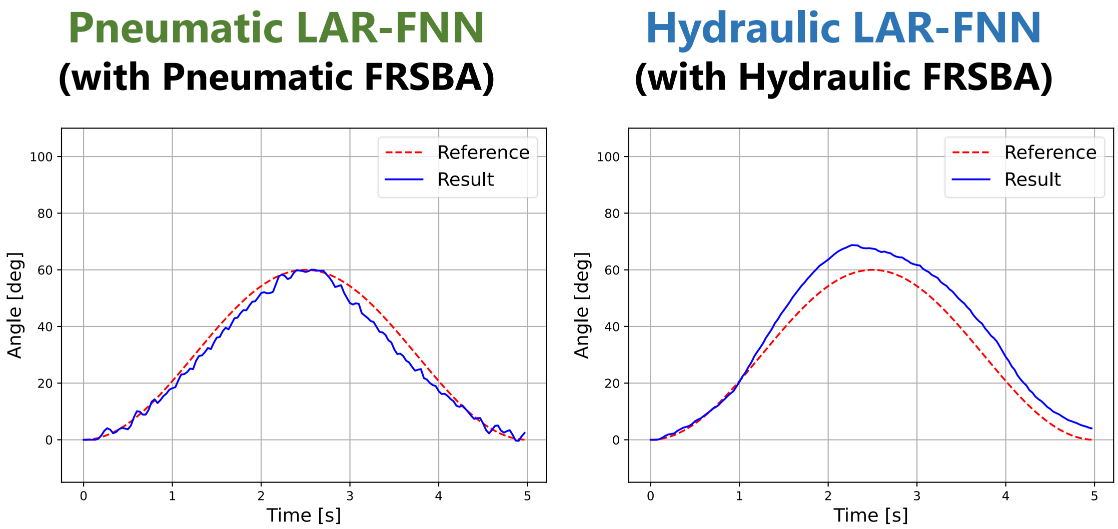

Figure S11. Control results with the Pneumatic LAR-FNN and the Hydraulic LAR-FNN for a single period sine trajectory with 0.2 Hz of frequency and 60 degrees of amplitude. The Pneumatic LAR-FNN controlled pneumatically actuated FRSBA No. 8 and the Hydraulic LAR-FNN controlled hydraulically actuated FRSBA No. 8. The experiment was conducted once.

Previous reseach on learning controllers for soft actuators

This section describes related research on learning controllers for soft actuators. Many researchers have utilized various machine learning methods (e.g., feedforward neural network,[1] reinforcement learning[2,3]) to model or control soft actuators,[4–6] dealing with their nonlinearity[7,8] and hysteresis.[9] Machine learning techniques are also already utilized for soft robot applications, such as a soft wearable device[10] and a soft manipulator.[11] The existing learning controllers can be classified into two types depending on the amount of prior model information: a model-based learning controller and a model-free learning controller.[12]

As an example of a model-based learning controller, which is based on prior model information, Tang *et al.* developed an iterative learning model predictive control.[13] This method needs just a simple, approximated model at the beginning. Then, an iterative learning controller (ILC) gradually improves and fits the initial model to the surrounding environments through a trial-and-error process. The controller achieved precise trajectory tracking in the experiments with a root mean square tracking error (RMSE) less than 0.03 rad at the 21st iteration. Note that another approach, like an adaptive scheme, also can be used for the model improvement.[14] Tang *et al.* proposed a probabilistic model-based online learning optimal control method. This method utilizes a probabilistic model, which is automatically derived as a Gaussian process, and the model is updated online with sensory data feedback during control. This controller achieved excellent control performance with low control RMSE and high robustness against disturbance.

As an example of a model-free learning controller which does not require prior information, Balasubramanian *et al.* proposed a PID controller with ILC for a soft rehabilitation device for upper limbs.[15] During the control, the ILC provides a model-free feedforward signal and improves its compatibility with the environment through iteration, i.e., trial and error. PID term provides a model-free feedback signal to enhance performance. This PID-with-ILC controller achieved high control precision with an absolute mean tracking error of less than 3°. Elgeneidy *et al.* proposed to use a feedforward neural network (FNN).[16] The researchers actuated a PSA to collect training data and trained the FNN to derive a model of a soft actuator and predict its bending angle. This method achieved high model accuracy. Likewise, Giorelli *et al.* used a neural network to obtain the inverse kinematics of a soft octopus-shaped manipulator.[17] Thuruthel *et al.* utilized a model-based reinforcement learning for closed-loop dynamic control of a soft manipulator with three actuate tendons.[18] As described in this paper's main text, we have also developed a model-free learning controller, an iterative learning-based feedforward neural network (IL-FNN). The IL-FNN was trained to represent an inverse model and achieved a precise tracking performance on various generalized trajectories with RMSE of 2.11°, compensating for soft actuators' individual deformability (individual characteristic differences) caused by the manual fabrication. Additionally, the IL-FNN achieved the individual deformability compensation of a hydraulic PSA, which previous research has not addressed.

As described in the main text, the existing learning control methods require a controller to be specialized to only one control target, and the re-specialization requires time. For example, the ILC used in the model-based learning controller[14] requires 21 times of iterations to modify the initial model again, which can take more than three minutes. Also, the neural network used in the model-free learning controller[16] needs 29,952 samples of training data at 100 Hz of the sampling

frequency, which takes about 5 minutes to be collected.

Selection of latent representation dimension and the architecture of the autoencoder

We first chose the dimension of a latent representation and then set the number of hidden layer neurons of the encoder and the decoder. Reffering the rare literature[12,19] that utilized an autoencoder in the field of soft robotics, we set the number of neurons of the encoder to be halved per layer toward the latent representation dimension from the fixed number of the input layer neurons of 900 (Fig. 1(A)). The decoder architecture was the mirror image of the encoder.

We trained the AE with the latent representation dimension of 2, 4, 8, 16, 32, 64, 128, and 256. Note that the corresponding number of encoder hidden layers to each dimension was 8, 7, 6, 5, 4, 3, 2, and 1, and the same training dataset described in section 3.3 was utilized. Fig. S10 shows train and validation loss for each latent representation dimension. The train and validation loss drastically increased when the latent representation dimension got less than 8, which means features of the FRSBAs were not sufficiently encoded.

A higher number of latent representation dimensions results in more input layer neurons of the FNN and more parameters and training time. Therefore, we selected 32 as the latent representation dimension, which had sufficiently converged losses and reliably encoded features of the FRSBAs with fewer number of dimension. Then, the number of layers and neurons of the encoder was determined based on the latent representation dimension, as in Fig. 1(A). Finally, the architecture of the decoder was determined to be the mirror image of the encoder.

Iterative learning controller

This section describes the detailed equation and the process flow of the Iterative Learning Controller (ILC), which is utilized to collect the training data for the Feedforward Neural Networks (FNN). Figure 2(B) shows the process flow of the ILC at the ith iteration. First, the ILC calculates the input $u_i(t)$ from the previous iteration data. Referring to the literature,[20] which proposed an iterative learning controller for a nonlinear time-delay repetitive system, we employed the simple model-free PD-type ILC for the LAR-FNN:

$$u_{i+1}(t) = \begin{cases} u_i(t) + \Gamma_1 e_i(t) + \Gamma_2 \dot{e}_i(t) & (i \neq 0) \\ 0 & (i = 0) \end{cases} \tag{S1}$$

where $e_i(t) \equiv \theta_\mathrm{d}(t) - \theta_i(t)$ is the error between a desired soft actuator output $\theta_\mathrm{d}(t)$ and a measured output $\theta_i(t)$, and $\dot{e}_i(t)$ is the time derivative of $e_i(t)$. $\dot{e}_i(t)$ is computed by the backward finite differences method after the calculation of $e_i(t)$. Γ_1, Γ_2 are the learning gains. Next, the conversion function converts $u_i(t)$ to $v_i(t) \equiv f(u_i(t))$. The soft actuator is controlled by $v_i(t)$. The conversion function linearizes the relation between u and a percentage of the FRSBA bending angle to the maximum angle (i.e., the bending angle when u is 100%). There are mainly three kinds of nonlinearities in the relationship between the PWM duty cycle and the FRSBA bending percentage: saturation, dead zone, and abrupt variation in the derivative of the relationship. The conversion function was utilized to make u and the FRSBA bending percentage equal value to reduce the nonlinearities. As a result, the conversion function improves the control performance of the ILC

and stabilizes the iterative learning process.[21] The $f(u)$ is designed for each soft actuator. $\theta_i(t)$ and $u_i(t)$ are saved in a memory of a control device (the "Memory" block in Fig. 2(B)) to use in the subsequent iteration. $\theta_i(t), v_i(t)$ are used for the training of the FNN.

In the experiments, learning gains were empirically set as $\Gamma_1 = 9.6, \Gamma_2 = 0.03$ for pneumatic actuation and $\Gamma_1 = 16.0, \Gamma_2 = 0.05$ for hydraulic actuation. As the input $u(t)$ was the percentage of the PWM duty cycle, $u(t)$ was limited, as shown in Equation (S2) before the value conversion.

$$u(t) = \begin{cases} 100 & (u(t) \geq 100) \\ u(t) & (0 < u(t) < 100) \\ 0 & (u(t) \leq 0) \end{cases} \tag{S2}$$

Note that before the calculation of $\dot{e}_i(t)$ in Equation (S1), $\theta_i(t)$ was processed with a discrete-time low-pass filter as shown in Equation (S3) where r was empirically set as $r = 0.99$ for both pneumatic and hydraulic actuation.

$$\theta_{\mathrm{LPF}}(t) = r\theta_i(t) + (1 - r)\theta_{\mathrm{LPF}}(t - 1) \tag{S3}$$

Then, $\theta_{\mathrm{LPF}}(t)$ was used for the calculation to reduce the noise. Also, the measured output $\theta_i(t)$ were processed with a low-pass filter with a cutoff frequency of 10 Hz after finishing all 15 iterations.

Operating principle and fabrication of a Fiber-Reinforced Soft Bending Actuator (FRSBA)

An FRSBA has a tubular structure with a semicircular-cross-section. Figure 3 in the reference[22] depicts the internal structure of an FRSBA. An elastomer forms an FRSBA structure, and reinforcements constrain the FRSBA expansion to create FRSBA's bending. Radial reinforcements (i.e., the radial strain limiting layer) limit the radial expansion. On the other hand, a strain limiting layer inhibits a flat surface extension. Thus, only the curved surface extends without radial expansion upon pressurization of the FRSBA. This uneven extension causes the FRSBA to bend. The detailed internal structure and fabrication process are described in the references.[22,23]

Rapid on/off switching of a three-port solenoid valve connected to an FRSBA (see Fig. 4) controls the FRSBA's internal pressure (i.e., the FRSBA's bending angle). The valve connects the FRSBA to the tank when it is off and connects the FRSBA to the pump when it is on (Fig. S9). When the valve is on, the pressurized fluid (air or water) flows into the FRSBA and increases the FRSBA internal pressure. On the contrary, when the valve is off, the elastic force of the pressurized FRSBA pushes its internal fluid to the tank, and the internal pressure decreases. In this way, the valve's rapid open/close switching with a 40 Hz PWM signal controls the fluids' flow into the FRSBA. Thus, the control input (PWM Duty Cycle, a ratio of the valve's on/off time per control period) can change the bending angle of the FRSBA.

In this paper, an FRSBA was fabricated with silicone with a shore hardness of 00–30 (Ecoflex 00-30, SmoothOn), thread as the radial reinforcements (Kevlar Yarn #30, ESCO), and a glass fiber tape as the strain limiting layer (Tiger G Fiber Tape N, Yoshino Gypsum). As described in the reference,[22] silicon casting and the addition of reinforcements produced the FRSBA.

References

[1] Gundula Runge, Mats Wiese, and Annika Raatz. Fem-based training of artificial neural networks for modular soft robots. In *IEEE ROBIO 2017*, pages 385–392, 2017.

[2] Haochong Zhang, Rongyun Cao, Shlomo Zilberstein, Feng Wu, and Xiaoping Chen. *Toward Effective Soft Robot Control via Reinforcement Learning*. Springer International Publishing, 2017.

[3] Sreeshankar Satheeshbabu, Naveen Kumar Uppalapati, Girish Chowdhary, and Girish Krishnan. Open loop position control of soft continuum arm using deep reinforcement learning. In *IEEE ICRA 2019*, pages 5133–5139, 2019.

[4] A. Melingui, R. Merzouki, J. B. Mbede, C. Escande, and N. Benoudjit. Neural networks based approach for inverse kinematic modeling of a compact bionic handling assistant trunk. In *IEEE ISIE 2014*, pages 1239–1244, 2014.

[5] Matthias Rolf and Jochen J. Steil. Efficient exploratory learning of inverse kinematics on a bionic elephant trunk. *IEEE Trans. Neural Netw. Learn. Syst.*, 25(6):1147–1160, 2013.

[6] Z. Q. Tang, H. L. Heung, K. Y. Tong, and Z. Li. A probabilistic model-based online learning optimal control algorithm for soft pneumatic actuators. *IEEE Rob. Autom. Lett.*, 5(2):1437–1444, 2020.

[7] Morgan T. Gillespie, Charles M. Best, Eric C. Townsend, David Wingate, and Marc D. Killpack. Learning nonlinear dynamic models of soft robots for model predictive control with neural networks. In *IEEE RoboSoft 2018*, pages 39–45, 2018.

[8] Phillip Hyatt, David Wingate, and Marc D. Killpack. Model-based control of soft actuators using learned non-linear discrete-time models. *Front. Robot. AI*, 6(22), 2019.

[9] Ying Zhang, Jinhai Gao, Hui Yang, and Lina Hao. A novel hysteresis modelling method with improved generalization capability for pneumatic artificial muscles. *Smart Mater. Struct.*, 28(10):105014, 2019.

[10] Brian Byunghyun Kang, Daekyum Kim, Hyungmin Choi, Useok Jeong, Kyu Bum Kim, Sungho Jo, and Kyu-Jin Cho. Learning-based fingertip force estimation for soft wearable hand robot with tendon-sheath mechanism. *IEEE Robot. Autom. Lett.*, 5(2):946–953, 2020.

[11] Juan M. Gandarias, Yongjing Wang, Agostino Stilli, Alfonso J. Garcia-Cerezo, Jesus M. Gomez de Gabriel, and Helge A. Wurdemann. Open-loop position control in collaborative, modular variable-stiffness-link (vsl) robots. *IEEE Robot. Autom. Lett.*, 5(2):1772–1779, 2020.

[12] Daekyum Kim, Sang-Hun Kim, Taekyoung Kim, Brian Byunghyun Kang, Minhyuk Lee, Wookeun Park, Subyeong Ku, DongWook Kim, Junghan Kwon, Hochang Lee, Joonbum Bae, Yong-Lae Park, Kyu-Jin Cho, and Sungho Jo. Review of machine learning methods in soft robotics. *PLOS ONE*, 16(2):e0246102, 2021.

[13] Z. Q. Tang, H. L. Heung, K. Y. Tong, and Z. Li. A novel iterative learning model predictive control method for soft bending actuators. In *IEEE Int. Conf. Rob. Autom.*, pages 4004–4010, 2019.

[14] Z. Q. Tang, H. L. Heung, K. Y. Tong, and Z. Li. Model-based online learning and adaptive control for a " human-wearable soft robot " integrated system. *Int. J. Rob. Res.*, 40(1):256–276, 2019.

[15] S. Balasubramanian, R. Wei, M. Perez, B. Shepard, E. Koeneman, and J. Koeneman. Rupert: an exoskeleton robot for assisting rehabilitation of arm functions. In *Virtual Rehab.*, pages 163–167, 2008.

[16] K. Elgeneidy, N. Lohse, and M. Jackson. Bending angle prediction and control of soft pneumatic actuators with embedded flex sensors – a data-driven approach. *Mechatronics*, 50:234–247, 2018.

[17] M. Giorelli, F. Renda, M. Calisti, A. Arienti, G. Ferri, and C. Laschi. Learning the inverse kinetics of an octopus-like manipulator in three-dimensional space. *Bioinspir. Biomim.*, 10(3):035006, 2015.

[18] Thomas G. Thuruthel, Egidio Falotico, Federico Renda, and Cecilia Laschi. Model-based reinforcement learning for closed-loop dynamic control of soft robotic manipulators. *IEEE Trans. Rob.*, 35(1):124–134, 2019.

[19] Marsela Polic, Ivona Krajacic, Nathan Lepora, and Matko Orsag. Convolutional autoencoder for feature extraction in tactile sensing. *IEEE Robot. Autom. Lett.*, 4(4):3671–3678, 2019.

[20] Hongfeng Tao, Hua Zhang, Huizhong Yang, and Jie Xu. Iterative learning control for nonlinear time-delay repetitive systems with arbitrary initial value. In *33rd Chinese Control Conference*, pages 8865–8870, 2014.

[21] Taku Sugiyama, Kyo Kutsuzawa, Dai Owaki, and Mitsuhiro Hayashibe. Individual deformability compensation of soft hydraulic actuators through iterative learning-based neural network. *Bioinpir. Biomim.*, 16(5):056016, 2021.

[22] P. Polygerinos, Z. Wang, K. C. Galloway, R. J. Wood, and C. J. Walsh. Soft robotic glove for combined assistance and at-home rehabilitation. *Rob. Auton. Syst.*, 73:135–143, 2015.

[23] Donal Holland, Conor J. Walsh, Max Herman, and Sara Berndt. soft robotics toolkit (accessed: 07/06/2022). `https://softroboticstoolkit.com/home`.
